# Clustering and Switching on verbal fluency tests in elementary school children with and without learning difficulties

**DOI:** 10.1590/2317-1782/20232022003en

**Published:** 2023-10-06

**Authors:** Diego Siqueira de Lima Teixeira, Maria Teresa Carthery-Goulart, Katerina Lukasova

**Affiliations:** 1 Programa de Pós-graduação em Neurociência e Cognição, Centro de Matemática, Computação e Cognição, Universidade Federal do ABC - UFABC - São Bernardo do Campo (SP), Brasil.

**Keywords:** Task Performance and Analysis, Underachievement, Academic Performance, Education Primary and Secondary, Neuropsychological Tests

## Abstract

**Purpose:**

Evaluate the performance in the Semantic and Phonemic Verbal Fluency tests in relation to the cognitive components of clustering and switching and explore the changes in development in elementary school.

**Methods:**

Participants were 68 children from the 2nd to 5th grade of elementary school of a public school in the municipality of Santo André, divided into two groups, Learning Difficulty (LD) and Typical Development (TD).

**Results:**

The Verbal Fluency tests were compared for the number of clusters, mean size of the clusters, and number of switches. All variables compared showed a statistically significant higher score for Semantic Verbal Fluency. Means and standard deviations of the same variables for year and group effect were realized in both Verbal Fluency tests. A statistically significant difference was observed only for the total number of clusters in the Semantic Verbal Fluency test for group effect, with the best performance of the TD group. A high correlation was observed between the total number of correct answers with the total number of clusters and number of switches in both Verbal Fluency tests. In addition, a correlation was observed between the total number of correct answers and the mean size of the clusters only in the Phonemic Verbal Fluency. Linear regression analysis showed greater variance for the total number of clusters, making it more predictable for performance in both verbal fluency tests.

**Conclusion:**

Verbal Fluency tests may be sensitive and predictive for the identification of possible differences in school performance associated with reading.

## INTRODUCTION

Verbal Fluency Tests (VFT) assess a complex set of cognitive processes related to the production of fluent speech, lexical access, word knowledge, and auditory attention^(1)^. Besides speech, VF works in reading comprehension, helping with lexical access. Since reading requires strategies for monitoring comprehension and making adjustments in case of incongruities, it is possibly also crucial for efficient access to information in successive order^(2,3)^.

VF can be measured using different cognitive tests. The most widely used are the phonemic verbal fluency (PVF) and the semantic verbal fluency (SVF) tests. Verbal Fluency (VF) tests were initially introduced to assess the global cognitive productivity of subjects with brain injuries, mainly frontal and temporal lobe injuries. They were later used by neuropsychology in the clinical field and research. Their assessment measure is sensitive to several cognitive functions and processes, such as memory, language, executive functions, and verbal aptitude. As they are easy and quick to apply, the VF tests have also been widely used in different age groups^(4,5)^.

Test content and application procedures may vary slightly. However, in general terms, participants are instructed to quickly recall words that begin with a letter (F, A, S, P, and so on) or belong to some semantic category (animals or fruits, for example). The time for evocations can range from 1 to 2 minutes, but 1 minute is usually a standard for application in adults. Some normative data are found in several languages, besides the expected performance by age group^(6)^.

Regarding executive control, processes such as monitoring, alternation, updating, and using new retrieval strategies are crucial for the retrieval of words in the PVF^(6,7,8,9)^. As for the evocation of words in the SVF, there is a high dependence on the cognitive operations of the lexical-semantic network, including linguistic representation, semantic knowledge, verbal knowledge, and lexical access^(7,10)^. Two cognitive components are employed as strategies for evoking words in the Verbal Fluency tests. The first can be evaluated through the total number and size of clusters, which measures the ability to recall words from the same semantic or orthographic subcategories related to semantic memory. The second component refers to the switch, the ability to change subcategories; therefore, related to cognitive flexibility and inhibitory control^(5,11,12)^.

Studies that assess the cognitive components of clustering and switching in a foreign language have increased and among international studies, two Israeli studies with healthy children found a developmental effect on clustering and switching scores^(13,14)^. The first study observed that the total number of clusters and switches increased significantly in both VF tests, which was not found in the mean size of clusters in the PVF test^(13)^. In the second study, the mean size of the clusters increased due to age, but only in the SVF test^(14)^. In summary, while the increase in VF indicates that it is related to continuous cognitive maturation, the second study emphasizes that this can be attributed to the development of more efficient executive search strategies, which can independently contribute more to the evocations of words than lexical maturation^(13,14)^.

In the national context, few Brazilian studies still focus on assessing the cognitive components of clustering and switching in children^(15)^.

One of the Brazilian studies compared the performance of clustering and switching variables in healthy children from public and private schools in the SVF and PVF tests. There was a better score in SVF than PVF and a difference in performance between age groups, and the 11-12 years old group had the highest number of clusters and switches. This result was indicative of the progressive maturation of executive functions^(16)^.

Another study^(12)^ standardized the methodology to analyze the clustering and switching cognitive components in the SVF and PVF tests for the Brazilian population^(5,11,17)^. In another study with healthy children with the standardization for the Brazilian population identified a different pattern of development in the VF tests (SVF and PVF) regarding the number of evocations and the clustering and switching components, with a higher prediction of the latter for performance in VF tests. Finally, the authors concluded that VF development depends on language, memory, and inhibitory control^(18)^.

Another study with a Brazilian sample evaluated two scoring method types for the clustering and switching components, namely, raw score and rates, the last one being the division of the variables total number of clusters, mean size of clusters, and the number of switches by the total number of evocations in the VF tests. In their sample with healthy children, differences were observed in the prediction results. In the raw scores analyses, the number of clusters, the size of the clusters, and the number of switches were predictors for the performance of the PVF test, while only the mean size of the clusters was a predictor in the rates analyses. The result favored the raw scores, which is the best evidence of validity^(19)^.

In a nutshell, there is a consensus that VF evolves with age differently in PVF and SVF, but the exact pattern of change in clustering and switching is still being investigated. The results of different studies corroborate in considering the SVF test easier than the PVF test, which indicates that other cognitive factors, such as cognitive effort and an active strategic search can affect VF differently throughout development^(4)^.

Thus, the present study aims to evaluate the performance in tests related to the clustering and switching cognitive components and which is the best predictive model for the performance in each VF test in second to fifth graders of elementary school with typical development and learning difficulties in a public school in the municipality of Santo André.

## METHODS

### Participants

Sixty-eight children of both sexes, aged 8-12 years, with age compatible with the school year, from the 2^nd^ to the 5^th^ year of elementary in a public school with Índice de Desenvolvimento da Educação Básica (IDEB) [Elementary Education Development Index] 5.5^(20)^ located in Santo André, participated in this project. The students were assessed from 2017 to 2019, and during that same period, some of the children were included in activities that complemented their reading program plan called Small-Step Learning to Read and Write (ALEPP)^(21)^. The responsible teachers indicated the inclusion due to the delayed acquisition of reading and writing, and this group was called the experimental group (learning difficulty - LD). Students without literacy delays were considered a control group (typical development - TD). Given the low number of participants in the LD group in the school years, school years were grouped to reduce the difference in the sample distribution. Students were grouped into two groups per school year (2^nd^ and 3^rd^ year and 4^th^ and 5^th^ year), as shown in Table 1. All parents or legal guardians signed the Informed Consent Term, and the students signed the Assent Term, approved by the Research Ethics Committee of the ABC Federal University under n° **2886946**.

**Table 1:** Total number of the sample by year and bundled group

**YEAR**	**LITERACY**				**TOTAL (F/M)**	
	**LD (F/M)**		**TD (F/M)**			
2 and 3	14	(5/9)	23	(15/8)	37	(20/17)
4 and 5	10	(5/5)	21	(9/12)	31	(14/17)
Total	24	(10/14)	44	(24/20)	68	(34/34)

Chi-squared analysis p = 0.05

**Caption:** LD = Learning Difficulty; TD = Typical Development; F = Female; M = Male

### Instruments and procedures

The evaluation was carried out individually by previously trained evaluators, lasting approximately 90 minutes in an isolated room provided by the school. All responses in the VF were recorded and later transcribed. The order of application was the same for all children. The SVF test was performed first, followed by the PVF test. In the SVF (animals) and PVF (letter P)^(22)^ tests, participants were asked to evoke words related to the letter P for a certain period (2 minutes) and later, words from the semantic category of animals. There is no maximum score in the VF tests since the maximum number of correct answers varies according to the total number of evocations performed in the stipulated period.

### Description of the PVF test clusters

Phonemic clusters are a set of successively generated words belonging to the same phonemic subcategories. In the PVF task, clusters are words that start with the same first two identical letters, rhymes, or are differentiated only by the vowel sound, keeping the first and last letters constant^(12)^.

### Description of the SVF test clusters

Semantic clusters were defined as the sets of words generated successively belonging to the same semantic subcategories presented in a study of the Brazilian sample, e.g., wild animals, aquatic animals, domestic animals, farm animals, birds, and insects^(12)^. Categories were assessed by independent judges to classify the animals not reported in the same study. Sixty-one students recruited by convenience among undergraduate and graduate students from the greater São Paulo region completed two online forms via the Google Forms platform. A total of 171 animals were classified and could be grouped by more than one characteristic to allow overlapping of the categories. For example, a “bee” has wings and could be categorized as an “animal with wings” and an “insect”. The forms raised the classification of the animals into six groups based on their characteristics, e.g., wild animal, domestic animal, farm animal, aquatic/semi-aquatic animal, winged animal, and insect.

### Data analysis

All the words evoked by the participants in each VF test were transcribed into an Excel spreadsheet in the order they were evoked. For the analysis of the VF tests, the total number of words evoked correctly were considered and, among the errors, words beginning with another letter, first names, state names (in the case of the PVF), and the other words that were not animal names (in the case of SVF). Moreover, repetitions, derivations of gender, and tense of the same word were also considered errors. Dependent variables were generated: the total number of clusters (sum of all clusters), the mean size of clusters (sum of words in each cluster from the second evoked word, divided by the child’s total number of clusters), and the number of switches (sum of the exchanges between the clusters, also considering the isolated words between the clusters). Descriptive analyses (mean and standard deviation) of the dependent variables and the comparison between subjects (TD and LD) and between school years (2^nd^ - 3^rd^ and 4^th^ - 5^th^ years) were performed. Subsequently, simple and multiple linear regression was performed to evaluate the best model for the dependent variables total number of clusters, mean size of clusters, and the number of switches, in the overall VF performance. The collected data were statistically processed using the Jamovi program, version 1.6.23, adopting a 0.05 significance level.

## RESULTS

**Figure 1:**
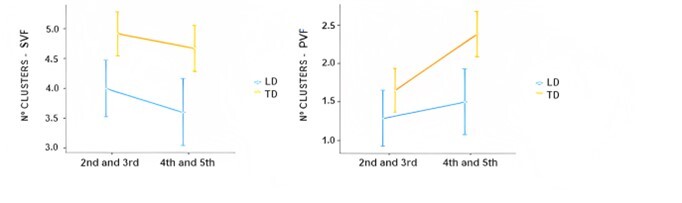
Total number of clusters in SVF (left) and PVF (right). Means were compared for LD and TD groups and school year. The scatter bar shows the standard error

The performance in the VF tests and in the variables total number of clusters, the mean size of clusters, and the number of switches for both VF tests (SVF and PVF) can be seen in Table 2. The effect of group and school years was compared using Kruskal-Wallis for the cognitive components of clustering and switching. Group comparisons concerning overall performance and other psychological metrics were described previously^(23)^. The variable total number of clusters showed better performance in the TD group compared to the LD group, according to a statistically significant difference for group effect in the SVF test [H (1) = 7.13; p=0.008], but there were no statistically significant differences in the PVF test, as shown in Figure 1. Furthermore, no statistically significant differences were found for the other variables, namely, mean cluster size and the number of switches in both VF tests.

**Table 2:** Means and standard deviations of performances in the SVF and PVF tests by groups and school years

	**DT**		**DA**	
	**2^nd^ and 3^rd^**	**4^th^ and 5^th^**	**2^nd^ and 3^th^**	**4^th^ and 5^th^**
	**(n = 23)**	**(n = 21)**	**(n = 14)**	**(n = 10)**
**SVF**				
Total correct answers	17,0 (4,2)	17,0 (4,2)	17,0 (4,2)	17,0 (4,2)
Nº Clusters	4,9 (2,0)	4,9 (2,0)	4,9 (2,0)	4,9 (2,0)
MS Clusters	1,9 (0,6)	1,9 (0,6)	1,9 (0,6)	1,9 (0,6)
Nº Switches	7,8 (2,6)	7,8 (2,6)	7,8 (2,6)	7,8 (2,6)
**PVF**				
Total correct answers	7,6 (3,5)	7,6 (3,5)	7,6 (3,5)	7,6 (3,5)
Nº Clusters	1,7 (1,3)	1,7 (1,3)	1,7 (1,3)	1,7 (1,3)
MS Clusters	1,2 (0,8)	1,2 (0,8)	1,2 (0,8)	1,2 (0,8)
Nº Switches	4,9 (2,3)	4,9 (2,3)	4,9 (2,3)	4,9 (2,3)

**Caption:** Nº Clusters = Total Number of Clusters; MS Clusters = Mean Size of Clusters; Nº Switches = Number of Switches

Correlations were analyzed for the entire sample without separation by group type. Spearman correlations were performed to assess the relationship between the total number of words produced in the PVF and SVF tests and the use of different strategies. The size of the correlation coefficient was interpreted per the values established as 0 - 0.29 = low correlation, 0.3 - 0.49 = medium, and 0.5 - 1.0 = high^(24)^. Only medium and high correlations will be reported below.

A high correlation was observed between the total number of correct answers, the number of clusters, and the number of switches in both VF tests. Moreover, a correlation was observed between the total number of correct answers and the mean size of the clusters only in the PVF, as shown in Table 3.

**Table 3:** Correlations between cluster and switch variables in VF tests

		T. Correct Answers	MS Clus	Nº Swit
PVF	Nº Clus	0.81***	0.61***	0.33**
	MS Clus	0.61***	-	NS
	Nº Swit	0.63***	-	-
SVF	Nº Clus	0.69***	0.30**	0.51***
	MS Clus	NS	-	-0.33**
	Nº Swit	0.75***	-	-

** p≤0.01

*** p≤0.001

**Caption:** T. Correct Answers = Total Correct Answers; Nº Clus = Total Number of Clusters; MS Clus = Mean Size of Clusters; Nº Swit = Number of Switches

Regressions were analyzed for the entire sample without separation by group type. Multiple linear regression was performed to evaluate the contributions of total clusters, the mean cluster size, and the number of switches in both VF tests. In the PVF test, the independent variables explain 88% of the performance variance. The Durbin-Watson fit index for this test was 1.87, while in the SVF test, the independent variables explain 74% of the performance variance (Table 4).

**Table 4:** Multiple linear regression for both VF tests

Variables	Β	ANOVA for the model	R^2^	Adjusted R^2^
*SVF*				
Model 1				
Total number of Clusters	0.77*	F (1.66) = 96.292*	0.61	0.62
Model 2				
Total number of Clusters	0.53*	F (2.65) = 97.742*	0.59	0.58
Number of Switches	0.46*			
Model 3				
Total number of Clusters	0.59*	F (3.64) = 110.840*	0.75	0.74
Number of Switches	0.55*			
Mean size of Clusters	0.33*			
*PVF*				
Model 1				
Total number of Clusters	0.78*	F (1.66) = 106.953*	0.62	0.61
Model 2				
Total number of Clusters	0.65*	F (2.65) = 119.397*	0.79	0.78
Number of Switches	0.43*			
Model 3				
Total number of Clusters	0.53*	F (3.64) = 163.703*	0.89	0.88
Number of Switches	0.47*			
Mean size of Clusters	0.34*			

* p = <.001

The Durbin-Watson index for multiple linear regression in the SVF test was 2.05. Therefore, simple linear regression analysis was performed to verify which independent variable was responsible for the index value in the SVF test. It was found that the mean cluster size was not the best predictor of performance in the SVF test (1%) (Table 5).

**Table 5:** Simple linear regression for both VF tests

Variables	Β	ANOVA for the model	R^2^
*SVF*			
Model 1			
Total number of Clusters	0.77*	F (1.66) = 96.3*	0.59
Model 2			
Number of Switches	0.74*	F (1.66) = 78.2*	0.54
Model 3			
Mean size of Clusters	-0.08	F (1.66) = 0.40	0.01
*PVF*			
Model 1			
Total number of Clusters	0.79*	F (1.66) = 107*	0.62
Model 2			
Number of Switches	0.63*	F (1.66) = 43.9*	0.40
Model 3			
Mean size of Clusters	0.50*	F (1.66) = 22.3*	0.25

* p = <.001

## DISCUSSION

This study presented the performance of public elementary school second to fifth graders in the semantic and phonemic verbal fluency tests for the cognitive components of clustering and switching. The comparison of tests showed better performance in SVF than in PVF concerning the total number of clusters, mean size of clusters, and the number of switches. When compared between school years and group (TD and LD students), only the total number of clusters was higher in the group of students with typical development, which is a significant difference only in the SVF test.

The differences between the tests regarding the total number of clusters, mean size of clusters, and switches in the SVF test were also found in a Brazilian study^(16)^ and the international literature^(13,14)^. In international studies, one study did not include the number of switches in the analysis. However, its results for the total number of clusters and cluster size were also more significant in the SVF test^(13)^. Another study showed a more significant number of clusters and switches for the SVF. However, the size of the clusters was more significant in the PVF test^(14)^. According to the literature, the PVF test presupposes more significant cognitive effort because it requires a very active strategic search^(4,25)^. Regarding the total number of clusters, we found a statistically significant group effect in the SVF test, indicating the better performance of the TD group. This result points to a temporal course of developing the PVF and SVF. The lexical-semantic access skills, established at around five years of age, and an effective search processing for this knowledge is the main factor in the SVF, while the development of strategic skills, such as alternation, are the main factors in the PVF^(5,16)^. Furthermore, the difference in performance observed in the TD group only in the SVF corroborates that this ability develops before the PVF and that the LD group may have had difficulty accessing lexical-semantic networks.

The correlations regarding the clustering and switching cognitive components with the total number of correct answers in the FV tests showed that both components are associated with the total number of correct answers in the SVF and PVF, suggesting that phonemic analysis, semantic categorization, and cognitive flexibility must be considered in the variability of the number of evocations produced^(5,11,26)^. In the present study, the total number of correct answers in the SVF test showed a positive correlation with the number of clusters and switches, which agrees with a national study in Brazilian Portuguese^(16)^ and other languages^(26,27)^.

The correlation between the total number of correct answers and the mean size of clusters varied between the tests. It was high in the PVF and only medium in the SVF. Studies in other non-anglophone languages have reached a similar result. A study with healthy adults fluent in German showed a positive correlation between the mean size of the clusters and the SVF test^(28)^. Moreover, two studies in the Hebrew language also showed a positive correlation between the size of the clusters and the total number of correct answers in the SVF^(13,14)^. It is believed that the linguistic-cultural differences between the different languages, including Brazilian Portuguese, may be responsible for this variation^(16)^.

The negative correlation of the number of switches with the mean size of clusters in the SVF test observed in the present study agrees with a Brazilian study^(16)^ and the international literature because in order to have a greater number of switches, it is necessary to reduce the size of the clusters^(11,26)^. This finding shows the need to balance these strategies already in childhood^(16)^. Moreover, the influence of the total number of clusters and mean size of clusters in the VF tests were identified, and concluded that the total number of clusters is more consistent than the size of the clusters due to 74% and 49% variance in the SVF test and PVF respectively^(13)^. This explanation agrees with the high correlation between the total number of correct answers and the total number of clusters observed in both tests in the present study.

The total number of correct answers in the PVF test showed a positive correlation with the total number of clusters, the mean size of clusters, and the number of switches. This result was also observed in the studies presented^(16,19,26,27)^. For good performance in the PVF test, “the components of executive functions, such as flexibility, strategic retrieval, and inhibition, are more important than those related to semantic memory and lexicon size”^(16:72)^.

Multiple linear regression indicated that the total number of clusters, the mean cluster size and the number of switches are predictors of performance in VF tasks (75% for SVF and 85% for PVF). The total number of clusters was highly predictive for the two VF tests, which agrees with other studies^(13,16)^. However, the variable mean size of clusters had lower predictive power, which is a result consistent with Brazilian Portuguese^(16,19)^ and Hebrew^(13)^ studies.

Although the highest percentage of variance in the number of switches was not observed in the present study, especially in PVF, another study showed a high variance (84%) of this cognitive component, suggesting that the switching strategy may be predominant in evoking words with phonological principles^(26)^. Switch is a component related to flexibility, evocation, and the use of strategies and inhibition, which are part of the executive functions with the peak of late maturation at 11 - 12 years of age^(18)^. We can only infer that these functions are poorly developed in the present study population. The effect of age and other cognitive functions, such as memory and language development, may have contributed to this result since the sample comprises children with typical development and learning difficulties^(26)^.

## CONCLUSION

The present study evaluated the cognitive components of clustering and switching in public school children with typical development and learning difficulties. It was observed that the clustering and switching components are essential strategies for analyzing VF tests. The variables showed statistically significant differences between the groups, with worse performance in students with learning difficulties only for the variable total number of clusters in the SVF test. Furthermore, they showed high variance in predicting performance in both VF tests and good tolerance to each other. The total number of clusters was the measure that best managed to predict the performance in the VF tests. However, the present and showcased studies revealed that cognitive flexibility, observed in the number of switches, associated with the total number of clusters might be the best strategy to predict performance in VF tests.

Most studies reported here were conducted with the child population with typical development at the national^(12,16,18,19)^ and international^(13,14,25,26,27)^, levels, suggesting the need for further studies to assess the development of VF in children with learning difficulties. This study has limitations. The TD and LD groups were not equally represented, which is the main limitation of this work. Moreover, another limitation of the study was to consider only the teachers’ referrals as a criterion for the LD group. Despite this, the study showed that it was possible to differentiate the performances in the VF tests between the LD and TD groups, however, only in the Semantic Fluency test for the variable total number of clusters, which is the only variable that differentiated children with and without learning difficulties.
