# Supplementary material for: Clustering and Switching on verbal fluency tests in elementary school children with and without learning difficulties
Source: Codas. 2023 Oct 6;35(6):e20220003. doi: 10.1590/2317-1782/20232022003en (PMC10702704; doi:10.1590/2317-1782/20232022003en)
Supplement: Translation

# Translation: *Clustering* e *Switching* nos testes de fluência verbal em crianças do ensino fundamental I com e sem dificuldade de aprendizagem

**DOI:** 10.1590/2317-1782/20232022003pt

**Authors:** Diego Siqueira de Lima Teixeira, Maria Teresa Carthery-Goulart, Katerina Lukasova

**Affiliations:** 1 Programa de Pós-graduação em Neurociência e Cognição, Centro de Matemática, Computação e Cognição, Universidade Federal do ABC - UFABC - São Bernardo do Campo (SP), Brasil.

**Keywords:** Análise e Desempenho de Tarefas, Baixo Rendimento Escolar, Desempenho Acadêmico, Ensino Fundamental e Médio, Testes Neuropsicológicos

## Abstract

**Objetivo:**

Avaliar o desempenho nos testes de Fluência Verbal Semântica e Fonêmica em relação aos componentes cognitivos de *clustering* e *switching* e explorar as mudanças no desenvolvimento no ensino fundamental.

**Método:**

Participaram 68 crianças do 2º ao 5º ano do ensino fundamental I de uma escola pública do município de Santo André, divididas em dois grupos, Dificuldade de Aprendizagem (DA) e Desenvolvimento Típico (DT).

**Resultados:**

Os testes de Fluência Verbal foram comparados para o número de *clusters*, tamanho médio dos *clusters* e número de *switches*. Todas as variáveis comparadas mostraram uma pontuação estatisticamente significante maior para o teste de Fluência Verbal Semântica. Foram realizadas as médias e desvios-padrão das mesmas variáveis para efeito de ano e grupo em ambos os testes. Foi observada diferença estatisticamente significante apenas para o número total de *clusters* no teste de Fluência Verbal Semântica para efeito de grupo, com o melhor desempenho do grupo DT. Observou-se correlação alta no total de acertos em ambos os testes de Fluência Verbal com o número total de *clusters* e número de *switches*. Além disso, foi observada correlação entre o total de acertos com a média de tamanho dos *clusters* apenas no teste de Fluência Verbal Fonêmica. A análise de regressão linear apresentou maior variância para o número total de *clusters,* o tornando mais preditivo para o desempenho em ambos testes de Fluência Verbal.

**Conclusão:**

Os testes de Fluência Verbal podem ser sensíveis e preditivos para a identificação de possíveis diferenças no desempenho escolar associados à leitura.

## INTRODUÇÃO

Os testes de Fluência verbal (TFV) avaliam um complexo conjunto de processos cognitivos relacionados com a produção da fala fluente, acesso lexical, conhecimento de palavras e atenção auditiva^(1)^. Além da fala, a FV atua na compreensão de leitura sendo auxiliar no acesso lexical e, uma vez que a leitura exige estratégias para monitoramento da compreensão e realização dos ajustes em caso das incongruências, possivelmente é importante também para o acesso eficiente das informações em ordem sucessiva^(2,3)^.

A FV pode ser medida por meio de diferentes testes cognitivos, sendo que os mais utilizados são teste de fluência verbal fonêmica (FVF) e teste de fluência verbal semântica (FVS). Os testes de Fluência Verbal (FV) foram inicialmente introduzidos como uma medida da avaliação da produtividade cognitiva global de sujeitos com lesões cerebrais, principalmente lesionados de lobo frontal e temporal. Posteriormente passaram a ser utilizados pela neuropsicologia também no campo clínico e nas pesquisas. Sua medida de avaliação é sensível a diversas funções e processos cognitivos como memória, linguagem, funções executivas e aptidão verbal. Por serem de fácil e rápida aplicação, os testes de FV têm sido bastante utilizados também em diferentes grupos etários^(4,5)^.

O conteúdo dos testes e os procedimentos da aplicação podem variar um pouco, mas em termos gerais, os participantes são instruídos a evocar rapidamente palavras que se iniciam com um tipo de letra (F, A, S, P etc.) ou que pertencem a alguma categoria semântica (animais, frutas e outros). O período para as evocações podem variar entre 1 a 2 minutos, mas 1 minuto costuma ser um padrão para a aplicação em adultos. Há dados normativos em diversas línguas, bem como o desempenho esperado por faixa etária^(6)^.

No que se refere ao controle executivo, processos como o monitoramento, alternância, atualização, além da utilização de novas estratégias de recuperação são considerados determinantes para a recuperação de palavras na FVF^(6,7,8,9)^. Já para a evocação de palavras na FVS, há alta dependência das operações cognitivas da rede léxico-semântica, dentre elas, representação linguística, conhecimento semântico, conhecimento verbal e acesso lexical^(7,10)^. Existem dois componentes cognitivos utilizados como estratégias nas evocações de palavras nos testes de Fluência Verbal. O primeiro pode ser avaliado por meio do número total e tamanho dos *clusters*, que mensura a habilidade da evocação de palavras das mesmas subcategorias semânticas ou ortográficas, relacionada à memória semântica. O segundo componente se refere ao *switch*, habilidade de troca de subcategorias, logo, relacionado à flexibilidade cognitiva e controle inibitório^(5,11,12)^.

Estudos que avaliam os componentes cognitivos de *clustering* e *switching* em língua estrangeira têm aumentado em número e entre os estudos internacionais, dois estudos israelenses com crianças saudáveis verificaram efeito de desenvolvimento nas pontuações de *clustering* e *switching* ^(13,14)^. O primeiro estudo observou que o número total de *clusters* e o número de *switches* aumentaram significativamente em ambos os testes de FV, mas o mesmo não foi encontrado no tamanho médio dos *clusters* no teste de FVF^(13)^. No segundo estudo o tamanho médio dos *clusters* aumentou em função da idade, mas apenas no teste de FVS^(14)^. Em resumo, enquanto o aumento na FV indica ser relacionado com uma contínua maturação cognitiva, o segundo estudo ressalta que isso pode ser atribuído ao desenvolvimento das estratégias executivas de buscas mais eficientes, que de forma independente, podem contribuir mais para as evocações de palavras do que a maturação lexical^(13,14)^.

No contexto nacional ainda são poucos os estudos brasileiros voltados para a avaliação dos componentes cognitivos de *clustering* e *switching* em crianças^(15)^.

Um dos estudos brasileiros comparou o desempenho das variáveis de *clustering* e *switching* em crianças saudáveis da escola pública e particular nos testes de FVS e FVF. Observou-se melhor pontuação em FVS do que a FVF e uma diferença no desempenho entre os grupos etários, sendo que o grupo de 11 e 12 anos apresentou o maior número de *clusters* e de *switches*. Esse resultado foi indicativo da progressiva maturação das funções executivas^(16)^.

Outro estudo^(12)^ realizou a padronização da metodologia para analisar os componentes cognitivos *clustering* e *switching* nos testes de FVS e FVF para a população brasileira^(5,11,17)^. Em outro estudo com crianças saudáveis com a padronização para a população brasileira, foi verificado que ocorre um padrão de desenvolvimento diferente nos testes de FV (FVS e FVF) no que diz respeito ao número de evocações e nos componentes de *clustering* e *switching*, ocorrendo uma maior predição do último para o desempenho nos testes de FV. Por fim, as autoras concluíram que o desenvolvimento da FV é mostrado depender da linguagem, memória e controle inibitório^(18)^.

Outro estudo com amostra brasileira avaliou dois tipos de método de pontuação para os componentes de *clustering* e switching, a saber, valor bruto e taxas, sendo a última, a divisão das variáveis número total de *clusters*, tamanho médio dos *clusters* e número de *switches* pelo número total de evocações nos testes de FV. Em sua amostra com crianças saudáveis, observou-se diferenças nos resultados de predição. Nas análises de valores brutos, o número de *clusters*, o tamanho dos *clusters* e o número de *switches*, foram preditores para o desempenho do teste de FVF, enquanto que nas análises de taxas, apenas o tamanho médio dos *clusters* foi preditor. O resultado favoreceu o uso da pontuação bruta tendo esta a melhor evidência de validade^(19)^.

Em resumo, há um consenso que a FV evolui com idade de forma diferente na FVF e na FVS, porém o exato padrão da mudança nos *clustering* e *switching* ainda é objeto de investigação. Os resultados de diferentes estudos corroboram em considerar o teste de FVS mais fácil que o teste de FVF, o que indica que outros fatores cognitivos, tais como esforço cognitivo e uma busca estratégica ativa podem afetar de forma distinta a FV ao longo do desenvolvimento^(4)^.

Portanto, o objetivo do presente estudo é avaliar o desempenho nos testes de relação aos componentes cognitivos de *clustering* e *switching* e, avaliar qual é o melhor modelo preditivo para o desempenho em cada um dos testes de FV em alunos do 2º ao 5º ano do ensino fundamental I com o desenvolvimento típico e com dificuldade de aprendizagem de uma escola pública do município de Santo André.

## MÉTODO

### Participantes

Nesse projeto participaram 68 crianças de ambos os sexos, com idades entre 8 - 12 anos, com idade compatível ao ano escolar, do 2º ano ao 5º ano do ensino infantil de uma escola pública com Índice de Desenvolvimento da Educação Básica (IDEB) 5.5^(20)^ localizada no município de Santo André. Os alunos passaram pela avaliação entre 2017 e 2019 e nesse mesmo período, algumas das crianças foram incluídas nas atividades complementares ao seu plano do programa de leitura chamado Aprendendo a Ler e Escrever em Pequenos Passos (ALEPP)^(21)^. A inclusão se deu pela indicação das professoras responsáveis, em função do atraso na aquisição da leitura e escrita, sendo esse grupo denominado como grupo experimental (dificuldade de aprendizagem - DA). Alunos sem atrasos na alfabetização foram considerados grupo controle (desenvolvimento típico - DT). Observando o baixo número de participantes do grupo DA nos anos escolares, optou-se em agrupar os anos escolares objetivando diminuir a diferença da distribuição amostral. Os alunos foram agrupados em dois grupos de acordo com ano escolar (2^o^ e 3^o^ ano e 4^o^ e 5^o^ ano) conforme Tabela 1. Todos os pais ou responsáveis legais assinaram Termo de Consentimento Livre e Esclarecido TCLE e os alunos assinaram o Termo de Assentimento, aprovados pelo Comitê de Ética e Pesquisa da Universidade Universidade Federal do ABC sob o nº **2886946**.

**Tabela 1:** Número total da amostra por ano e grupo agrupados

**ANO**	**ALFABETIZAÇÃO**				**TOTAL (F/M)**	
	**DA (F/M)**		**DT (F/M)**			
2 e 3	14	(5/9)	23	(15/8)	37	(20/17)
4 e 5	10	(5/5)	21	(9/12)	31	(14/17)
Total	24	(10/14)	44	(24/20)	68	(34/34)

Análise com Qui-quadrado p = 0,05

**Legenda:** DA = Dificuldade de Aprendizagem; DT = Desenvolvimento Típico; F = Feminino; M = Masculino

### Instrumentos e procedimentos

A avaliação foi realizada individualmente por avaliadores previamente treinados com duração de aproximadamente 90 minutos numa sala isolada disponibilizada pela escola. Todas as respostas no FV foram gravadas e transcritas posteriormente. A ordem de aplicação foi a mesma para todas as crianças, sendo primeiramente realizado o teste de FVS e logo em seguida, o teste de FVF. Nos testes de FVS (animais) e FVF (letra P)^(22)^ foi solicitado que durante um determinado período de tempo (2 minutos) os participantes evocassem palavras, relacionadas à letra P e, num outro momento, palavras da categoria semântica de animais. Não há pontuação máxima nos testes de FV, pois, o máximo de acerto varia de acordo com o total de evocações realizadas no período estipulado.

### Descrição dos *clusters* no teste de FVF

*Clusters* fonêmicos são considerados um conjunto de palavras geradas sucessivamente que pertencem às mesmas subcategorias fonêmicas. Na tarefa de FVF, os *clusters* são considerados palavras que iniciam com as mesmas duas primeiras letras iguais, rimas ou que se diferenciam apenas pelo som da vogal, mantendo as primeiras e últimas letras constantes^(12)^.

### Descrição dos *clusters* no teste de FVS

Foram considerados *clusters* semânticos os conjuntos de palavras geradas sucessivamente pertencentes às mesmas subcategorias semânticas apresentadas em um estudo de com a amostra brasileira, e.g. animais selvagens, animais aquáticos, animais de ambiente doméstico, animais de criação, aves e insetos^(12)^. A fim de classificar os animais não relatados no mesmo estudo, foi realizada uma avaliação própria de categorias com juízes independentes. Os preenchimentos de 02 formulários online foram realizados via plataforma Google Forms por 61 estudantes recrutados por conveniência entre estudantes de graduação e de pós-graduação da região da grande São Paulo. Um total de 171 animais foram classificados podendo ser agrupados por mais de uma característica a fim de permitir sobreposição das categorias. Por exemplo, “abelha” tendo asas, poderia ser categorizada como “animal com asas” e “inseto”. Os formulários levantaram as formas de classificação dos animais em 06 agrupamentos baseados em suas características i. e. Animal selvagem, animal doméstico, animal de criação, animal aquático / semiaquático, animal com asas e insetos.

### Análise dos dados

Transcreveram-se em planilha Excel todas as palavras evocadas pelos participantes em cada teste de FV, na ordem em que foram evocadas. Para a análise dos testes de FV, foram considerados o número total de palavras evocadas corretamente e entre os erros, foram computadas palavras iniciadas por outra letra, nomes próprios, nomes de estados (no caso da FVF) e, as demais palavras que não eram nomes de animais (no caso da FVS). Além disso, as repetições, sendo derivações de gênero e tempo verbal da mesma palavra, também foram consideradas erros. Foram geradas variáveis dependentes: o número total de *clusters* (soma de todos *clusters*), tamanho médio dos *clusters* (soma das palavras de cada *cluster* a partir da segunda palavra evocada, dividido pelo número total de *cluster* da criança) e número de *switches* (soma das trocas entre os *clusters* considerando-se também as palavras isoladas entre os *clusters*).

Foram realizadas análises descritivas (média e desvio-padrão) das variáveis dependentes e comparação entre sujeitos (DT e DA) e entre ano escolar (2^o^ - 3^o^ ano e 4^o^ - 5^o^). Posteriormente, foi realizada regressão linear simples e múltipla a fim de se avaliar o melhor modelo para as variáveis dependentes número total de *clusters*, tamanho médio dos *clusters* e número de *switches,* no desempenho geral na FV. Os dados recolhidos foram tratados estatisticamente por meio do programa Jamovi versão 1.6.23 e o nível de significância adotado foi de 0,05.

## RESULTADOS

O desempenho nos testes de FV e nas variáveis número total de *clusters*; tamanho médio dos *clusters* e número de *switches,* para ambos os testes de FV (FVS e FVF) podem ser observados na Tabela 2. Foram comparados o efeito de grupo e anos escolares por meio de Kruskal-Wallis para os componentes cognitivos de *clustering* e *switching*. As comparações dos grupos em relação ao desempenho geral e outras métricas psicológicas foram descritos anteriormente^(23)^. A variável número total de *clusters,* apresentou melhor desempenho do grupo DT em comparação com o grupo DA, conforme diferença estatisticamente significante para efeito de grupo no teste de FVS [H (1) = 7,13; p = 0,008], mas não ocorreram diferenças estaticamente significantes no teste de FVF, conforme Figura 1. Além disso, não foram encontradas diferenças estatisticamente significantes para as demais variáveis, a saber, tamanho médio dos *clusters* e número de *switches* em ambos os testes de FV.

**Tabela 2:** Médias e desvios-padrão dos desempenhos nos testes de FVS e FVF por grupos e anos escolares

	**DT**		**DA**	
	**2º e 3º**	**4º e 5º**	**2º e 3º**	**4º e 5º**
	**(n = 23)**	**(n = 21)**	**(n = 14)**	**(n = 10)**
**FVS**				
Total de acertos	17,0 (4,2)	18,3 (4,5)	16,0 (7,0)	16,2 (4,0)
Nº *Clusters*	4,9 (2,0)	4,7 (1,7)	4,0 (1,8)	3,6 (1,1)
MT *Clusters*	1,9 (0,6)	2,3 (1,8)	1,9 (0,6)	2,2 (0,9)
Nº *Switches*	7,8 (2,6)	8,8 (3,3)	7,6 (3,7)	7,7 (3,6)
**FVF**				
Total de acertos	7,6 (3,5)	11,4 (5,0)	7,6 (4,7)	7,7 (4,2)
Nº *Clusters*	1,7 (1,3)	2,4 (1,5)	1,3 (1,1)	1,5 (1,4)
MT *Clusters*	1,2 (0,8)	1,5 (1,3)	1,3 (1,3)	1,8 (1,4)
Nº *Switches*	4,9 (2,3)	6,9 (3,3)	5,6 (2,8)	5,1 (3,3)

**Legenda:** Nº *Clusters* = Número Total de *Clusters*; MT *Clusters* = Média de Tamanho dos *Clusters*; Nº *Switches* = Número de *Switches*

**Figura 1:** Número total de *clusters* na FVS (à esquerda) e FVF (à direita). As médias foram comparadas para grupo DA e DT e ano escolar. A barra de dispersão mostra o erro padrão

As correlações foram analisadas para amostra inteira sem separação por tipo de grupo. Realizaram-se correlações de Spearman para avaliar a relação entre o número total de palavras produzidas nos testes de FVF e FVS e o uso de diferentes estratégias. A magnitude do coeficiente de correlação foi interpretada de acordo com os valores estabelecidos como 0 a 0,29 = correlação baixa; 0,3 a 0,49 = média e 0,5 a 1,0 = alta^(24)^. A seguir serão reportadas apenas correlações médias e altas.

Observou-se correlação alta no total de acertos com o número total de *clusters* e número de *switches* em ambos os testes de FV. Além disso, foi observada correlação entre o total de acerto com a média de tamanho dos *clusters* apenas na FVF, conforme Tabela 3.

**Tabela 3:** Correlações entre as variáveis *cluster* e *switch* nos testes de FV

		T. Acertos	MT Clus	Nº Swit
FVF	Nº Clus	0,81***	0,61***	0,33**
	MT Clus	0,61***	-	NS
	Nº Swit	0,63***	-	-
FVS	Nº Clus	0,69***	0,30**	0,51***
	MT Clus	NS	-	-0,33**
	Nº Swit	0,75***	-	-

** p≤0,01

*** p≤0,001

**Legenda:** T. Acertos = Total de Acertos; Nº Clus = Número Total de *Clusters*; MT Clus = Média de Tamanho dos *Clusters*; Nº Swit = Número de *Switches*

As regressões foram analisadas para amostra inteira sem separação por tipo de grupo. Foi realizada regressão linear múltipla para avaliar as contribuições do total de *clusters*, tamanho médio dos *clusters* e número de *switches* em ambos os testes de FV. No teste de FVF as variáveis independentes explicam 88% da variância no desempenho. O índice de ajuste Durbin-Watson para este teste foi 1,87 enquanto que no teste de FVS as variáveis independentes explicam 74% da variância no desempenho (Tabela 4).

**Tabela 4:** Regressão linear múltipla para ambos os testes de FV

Variáveis	Β	ANOVA para modelo	R^2^	R^2^ Ajustado
*FVS*				
Modelo 1				
Número Total de *Clusters*	0,77*	F (1,66) = 96.292*	0,61	0,62
Modelo 2				
Número Total de *Clusters*	0,53*	F (2,65) = 97.742*	0,59	0,58
Número de *Switches*	0,46*			
Modelo 3				
Número Total de *Clusters*	0,59*	F (3,64) = 110.840*	0,75	0,74
Número de *Switches*	0,55*			
Tamanho Médio dos *Clusters*	0,33*			
*FVF*				
Modelo 1				
Número Total de *Clusters*	0,78*	F (1,66) = 106.953*	0,62	0,61
Modelo 2				
Número Total de *Clusters*	0,65*	F (2,65) = 119.397*	0,79	0,78
Número de *Switches*	0,43*			
Modelo 3				
Número Total de *Clusters*	0,53*	F (3,64) = 163.703*	0,89	0,88
Número de *Switches*	0,47*			
Tamanho Médio dos *Clusters*	0,34*			

* p = <,001

O índice de Durbin-Watson para a regressão linear múltipla no teste de FVS foi 2,05. Portanto, foi realizada análise de regressão linear simples, para verificar qual variável independente estava sendo responsável pelo valor do índice no teste de FVS. Foi verificado que a variável tamanho médio dos *clusters* não foi a melhor variável para predizer o desempenho no teste de FVS (1%) (Tabela 5).

**Tabela 5:** Regressão linear simples para ambos os testes de FV

Variáveis	Β	ANOVA para modelo	R^2^
*FVS*			
Modelo 1			
Número Total de *Clusters*	0,77*	F (1,66) = 96.3*	0,59
Modelo 2			
Número de *Switches*	0,74*	F (1,66) = 78.2*	0,54
Modelo 3			
Tamanho Médio dos *Clusters*	-0,08	F (1,66) = 0.40	0,01
*FVF*			
Modelo 1			
Número Total de *Clusters*	0,79*	F (1,66) = 107*	0,62
Modelo 2			
Número de *Switches*	0,63*	F (1,66) = 43.9*	0,40
Modelo 3			
Tamanho Médio dos *Clusters*	0,50*	F (1,66) = 22.3*	0,25

* p = <,001

## DISCUSSÃO

Este estudo apresentou o desempenho das crianças do 2º ao 5º ano do ensino fundamental da rede pública nos testes de fluência verbal semântica e fonêmica para os componentes cognitivos de *clustering* e *switching*. A comparação dos testes mostrou melhor desempenho na FVS do que em FVF no número total de *clusters,* tamanho médio dos *clusters* e número de *switches.* Quando comparado entre anos escolares e grupo (alunos DT e DA) apenas o número total de *clusters* foi maior no grupo de alunos com desenvolvimento típico, sendo isso uma diferença significante apenas no teste FVS.

As diferenças entre os testes em relação ao número total de *clusters*, tamanho médio dos *clusters* e número de *switches* no teste de FVS foi também encontrada em estudo brasileiro^(16)^ e na literatura internacional^(13,14)^. Nos estudos internacionais, um estudo não incluiu o número de *switches* na análise, mas seus resultados do número total de *clusters* e tamanho dos *clusters* também foram maiores no teste de FVS^(13)^. Outro estudo apresentou maior número de *clusters* e *switches* para a FVS, porém, o tamanho dos clusters foi maior no teste de FVF^(14)^. De acordo com a literatura, o teste de FVF exige maior esforço cognitivo por exigir uma busca estratégica mais ativa^(4,25)^. Em relação ao número total de *clusters,* encontramos um efeito de grupo estatisticamente significante no teste de FVS, indicando melhor desempenho do grupo DT. Esse resultado aponta para um curso temporal de desenvolvimento da FVF e FVS, sendo que as habilidades de acesso léxico-semântico, que começam a se estabelecer por volta dos 05 anos de idade, bem como um processamento de busca eficaz a esse conhecimento é principal fator na FVS, enquanto que o desenvolvimento de habilidades estratégicas, como alternância, são fatores principais na FVF^(5,16)^. Além disso, a diferença de desempenho observada no grupo DT apenas na FVS corrobora que esta habilidade se desenvolve anteriormente à FVF, e que o grupo DA pode ter tido dificuldade no acesso às redes léxico-semânticas.

As correlações apresentadas em relação aos componentes cognitivos *clustering* e *switching* com o total de acertos nos testes de FV mostraram que ambos componentes estão associados ao número total de acertos tanto na FVS quanto na FVF, sugerindo que tanto a análise fonêmica, categorização semântica e, a flexibilidade cognitiva, devem ser levadas em consideração na variabilidade do número de evocações produzidas^(5,11,26)^. No presente estudo, o total de acertos no teste de FVS apresentou correlação positiva com o número total de *clusters* e número de *switches,* o que está de acordo com um estudo nacional no idioma português brasileiro^(16)^ e também de outras línguas^(26,27)^.

A correlação entre o total de acertos e tamanho médio dos *clusters* variou entre os testes, sendo alta na FVF e apenas média na FVS. Estudos em outras línguas, que não pertencem entre as anglófonas, chegaram ao resultado similar. Um estudo com adultos saudáveis fluentes no idioma alemão apresentou correlação positiva entre o tamanho médio dos *clusters* e o teste de FVS^(28)^. Além disso, dois estudos no idioma hebraico também apresentaram correlação positiva entre o tamanho dos *clusters* com o total de acertos na FVS^(13,14)^. Acredita-se que as diferenças linguístico-culturais dos diferentes idiomas incluindo o Português brasileiro podem ser responsáveis por essa variação^(16)^.

A correlação negativa do número de *switches* com o tamanho médio dos *clusters* no teste de FVS observada no presente estudo está de acordo com um estudo brasileiro^(16)^ e na literatura internacional, pois para que se ocorra um número maior de *switches*, é necessário que se diminua o tamanho dos *clusters* ^(11,26)^. O que demonstra a necessidade de um balanceamento dessas estratégias já na infância^(16)^. Além disso, foi verificado a influência do número total de *clusters* e tamanho médio dos *clusters* nos testes de FV e concluíram que o número total de *cluster* é mais consistente que o tamanho dos *clusters* devido 74% e 49% da variância desse no teste de FVS e FVF respectivamente^(13)^. Essa explicação está de acordo com a correlação alta para o total de acertos e número total de *clusters* observada em ambos os testes no presente estudo.

O total de acertos no teste de FVF apresentou correlação positiva com o número total de *clusters*, tamanho médio dos *clusters* e número de *switches*, esse resultado também foi observado nos estudos apresentados^(16,19,26,27)^. Para o bom desempenho no teste de FVF, “os componentes das funções executivas de flexibilidade, recuperação estratégica e inibição, são mais importantes que os relacionados à memória semântica e tamanho do léxico”^(16:72)^.

A regressão linear múltipla indicou que o número total de *clusters*, tamanho médio dos *clusters* e número de *switches* são preditores do desempenho nas tarefas de FV (75% para FVS e 85% para FVF). O número total de *clusters* mostrou-se ser mais preditivo para os dois testes de Fluência Verbal, o que está de acordo com outros estudos^(16,13)^, porém a variável tamanho médio dos *clusters* obteve menor poder preditivo, sendo esse um resultado coerente com os estudos em português brasileiro^(16,19)^ e no idioma hebraico^(13)^.

Ainda que não se tenha observado a maior porcentagem da variância no número de switches no presente estudo, principalmente na FVF, outro estudo mostrou alta variância (84%) desse componente cognitivo, sugerindo que a estratégia de switching possa ser predominante nas evocações de palavras com princípios fonológicos^(26)^. *Switch* é um componente relacionado com flexibilidade, evocação e com uso de estratégias e inibição que fazem parte das funções executivas com o pico de maturação tardia aos 11 - 12 anos^(18)^. Podemos apenas inferir que essas funções estão pouco desenvolvidas na população do presente estudo. O efeito de idade e outras funções cognitivas, tais como memória e o desenvolvimento da linguagem em geral podem ter contribuído com esse resultado uma vez que a amostra está composta pelas crianças com desenvolvimento típico e com dificuldade de aprendizagem^(26)^.

## CONCLUSÃO

No presente estudo foram avaliados os componentes cognitivos de *clustering* e *switching* em crianças da escola pública com desenvolvimento típico e com dificuldade de aprendizagem. Foi observado que os componentes de *clustering* e s*witching*, são importantes estratégias para a análise nos testes de FV. As variáveis apresentaram diferenças estatisticamente significante entre os grupos, com pior desempenho em alunos com dificuldade de aprendizagem apenas para a variável número total de *clusters* no teste de FVS. Além disso, apresentaram alta variância para predizer o desempenho em ambos os testes de FV, e boa tolerância entre si. O número total de *clusters* foi a medida que melhor conseguiu predizer o desempenho nos testes de FV, embora foi observado no presente estudos e nos apresentados, que a flexibilidade cognitiva, observada no número de *switches* associada ao número total de *clusters* pode ser a melhor estratégia para predizer o desempenho nos testes de FV.

A maioria dos estudos aqui relatados foram realizados com a população infantil com desenvolvimento típico no nível nacional^(12,16,18,19)^ e internacional^(13,14,25,26,27)^, sugerindo a necessidade de novos estudos que avaliem o desenvolvimento da FV na população infantil com dificuldade de aprendizagem. Este estudo possui limitações. Os grupos DT e DA, não foram igualmente representados, o que configura limitação principal deste trabalho. Além disso, outra limitação do estudo foi considerar apenas os encaminhamentos das professoras como critério para o grupo DA. Apesar disso, o estudo mostrou que foi possível diferenciar os desempenhos nos testes de FV entre os grupos DA e DT, porém, apenas no teste de Fluência Verbal Semântica para a variável número total de *clusters*. Sendo a única variável que se mostrou capaz de diferenciar crianças com e sem dificuldade de aprendizagem.
